# Supplementary material for: Using multiple sampling strategies to estimate SARS-CoV-2 epidemiological parameters from genomic sequencing data
Source: Nat Commun. 2022 Sep 23;13:5587. doi: 10.1038/s41467-022-32812-0 (PMC9508174; doi:10.1038/s41467-022-32812-0)
Supplement: Supplementary file 1 — Supplementary Information [file 41467_2022_32812_MOESM1_ESM.pdf]

Supplementary Figures and Tables

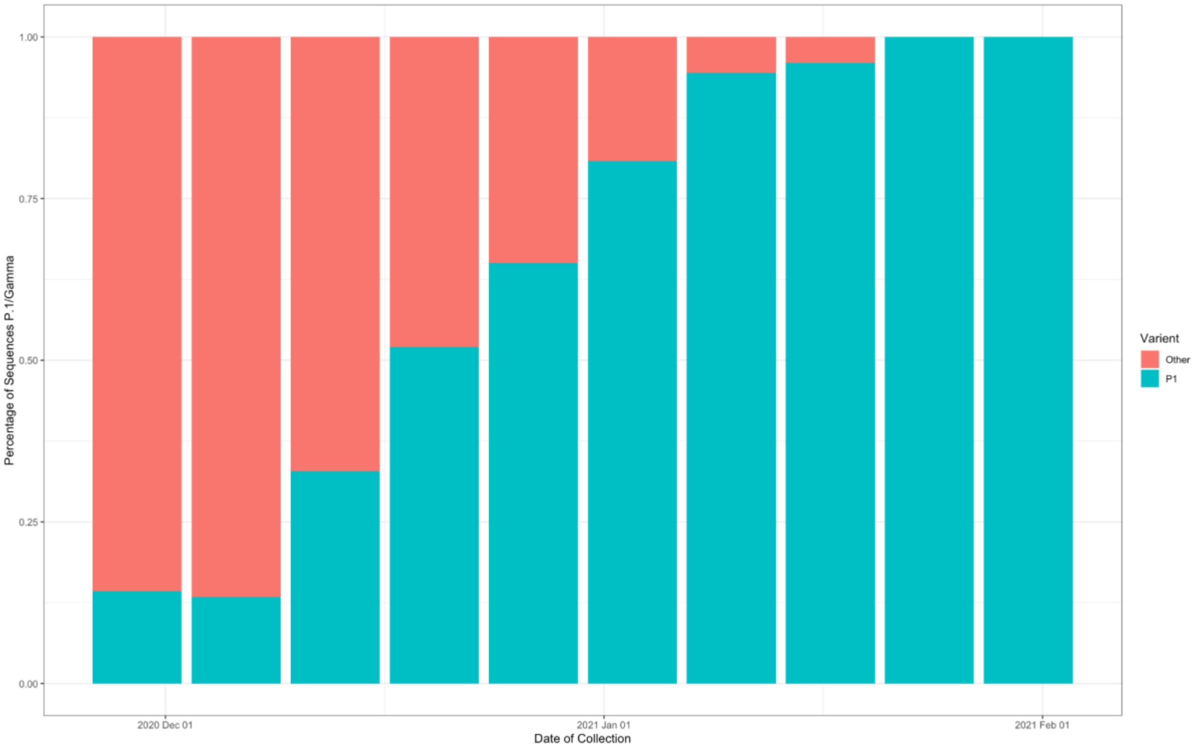

Supplementary Figure 1: The proportion of P.1 sequences compared to non-P.1 sequences from Amazonas State, Brazil found on GISaid (Shu and McCauley, 2017).

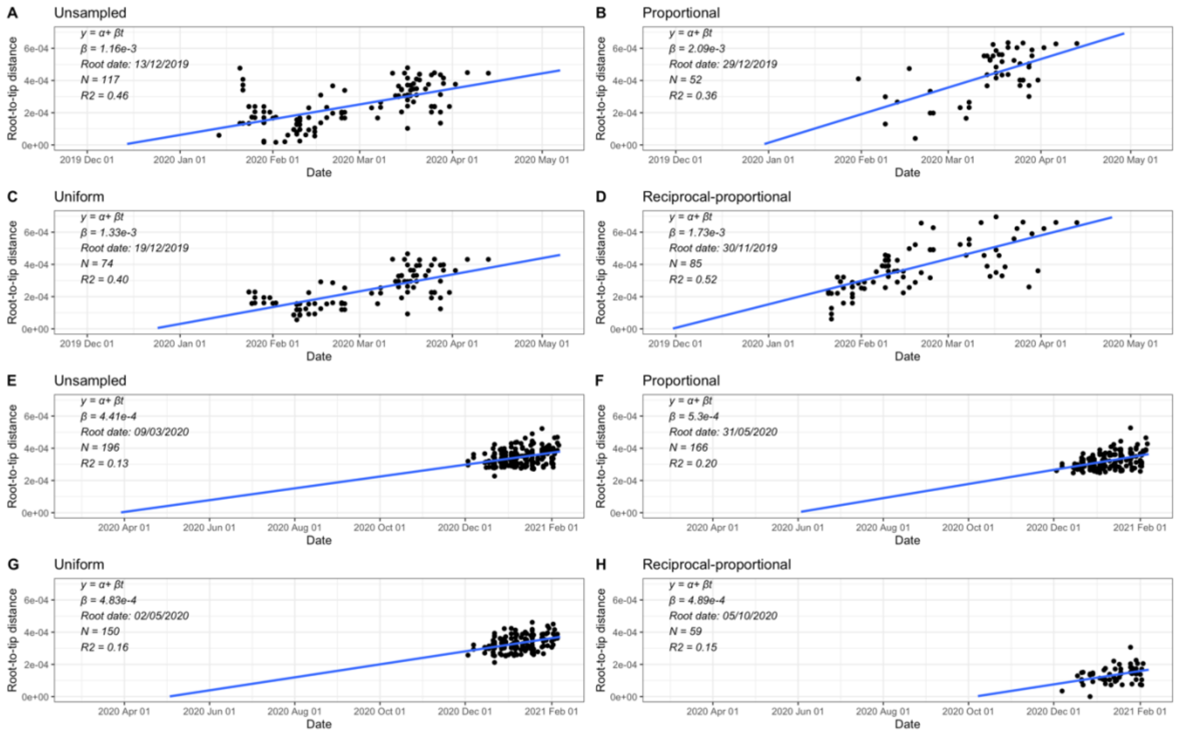

Supplementary Figure 2: Root-to-tip genetic distances to sample collection dates for the SARS-CoV-2 genome datasets used in this study: A-D represents Hong Kong and E-H represent Amazonas State. Plots are based on the maximum likelihood trees rooted by maximising R^2. The linear regression trend lines are shown to data points, corresponding to the genome sequences (represented with black dots).

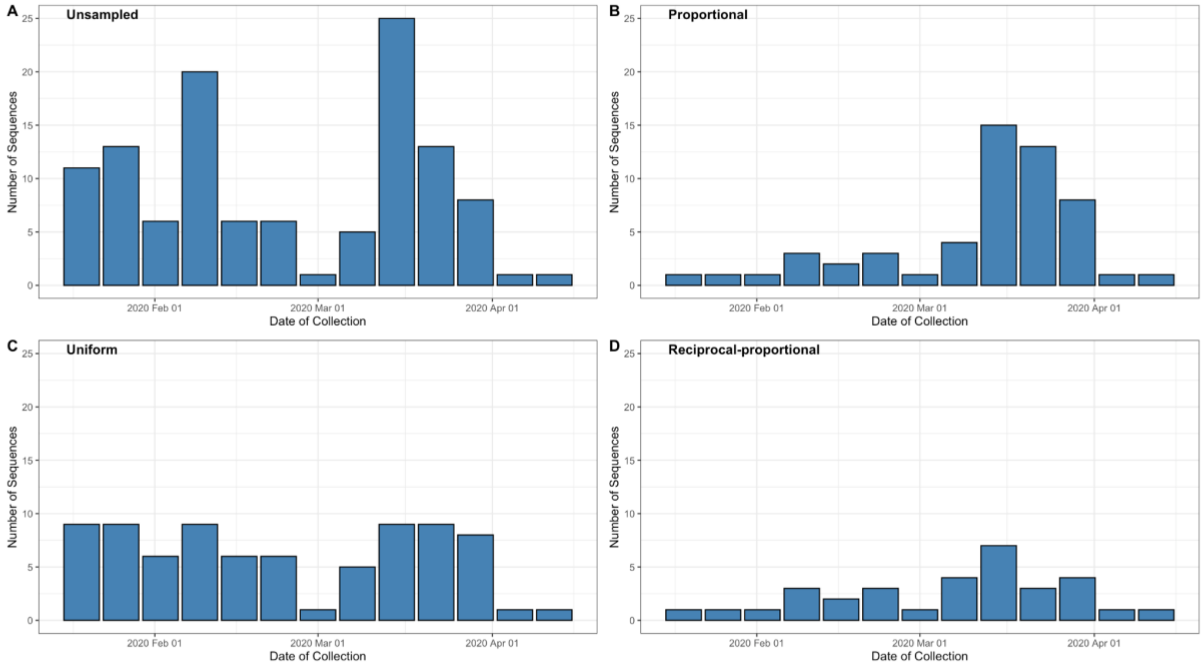

Supplementary Figure 3: Number of sequences for each week and sampling scheme for Hong Kong dataset.

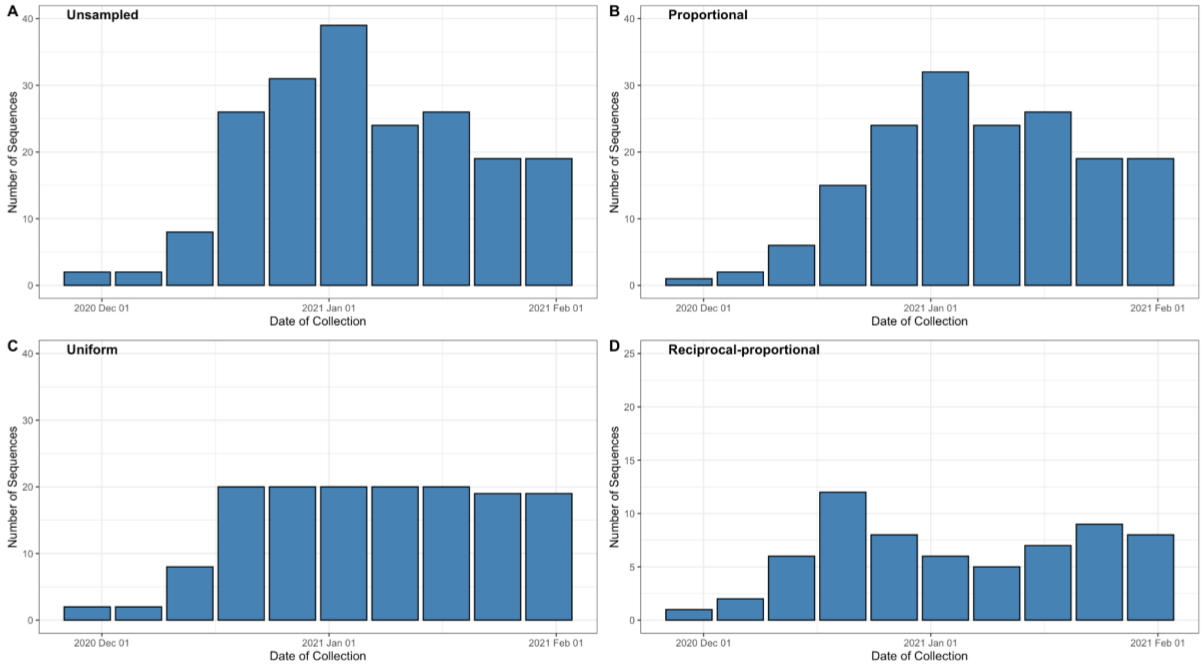

Supplementary Figure 4: Number of sequences for each week and sampling scheme for Amazonas dataset.

Supplementary Table 1: TMRCA and mean substitution rate both with 95% BCI for each sampling strategy for Hong Kong and Amazonas datasets alongside the Jensen-Shannon distance. Full posterior distribution of the TMRCA and substitution rates obtained under the different sampling strategies can be found in Figure 3B and D and Supplementary Figure 5.

**Sampling Strategy**	**Dataset**	**TMRCA (95% BCI)**	**Mean Substitution Rate (95% BCI, subs/site/year, s/s/y)**
Unsampled	Hong Kong	2nd December 2019 (10th November 2019 – 24th December 2019)	1.12×10^{-3} (9.16×10^{-4} – 1.35×10^{-3})
	Brazil	30th October 2020 (8th October 2020 – 13th December 2020)	4.58×10^{-4} (3.69×10^{-4} – 5.56×10^{-4})
Proportional	Hong Kong	24th December 2019 (21st November 2019 – 11th January 2020)	1.39×10^{-3} (9.28×10^{-4} – 2.48×10^{-3})
	Brazil	30th October 2020 (25th August 2020 – 29th November 2020)	4.60×10^{-4} (3.70×10^{-4} – 5.56×10^{-4})
Uniform	Hong Kong	13th December 2019 (18th November 2019 – 4th January 2020)	1.64×10^{-3} (1.22×10^{-3} – 2.09×10^{-3})

	Brazil	27th October 2020 (5th October 2020 – 25th November 2020)	$4.60x10^{-4}$ ($3.70x10^{-4}$ – $5.56x10^{-4}$)
Reciprocal-proportional	Hong Kong	6th December 2019 (10th November 2019 – 28th December 2019)	$1.30x10^{-3}$ ($1.03x10^{-3}$ – $1.59x10^{-3}$)
	Brazil	30th October 2020 (27th September 2020 – 25th November 2020)	$4.00x10^{-4}$ ($2.56x10^{-4}$ – $5.55x10^{-4}$)

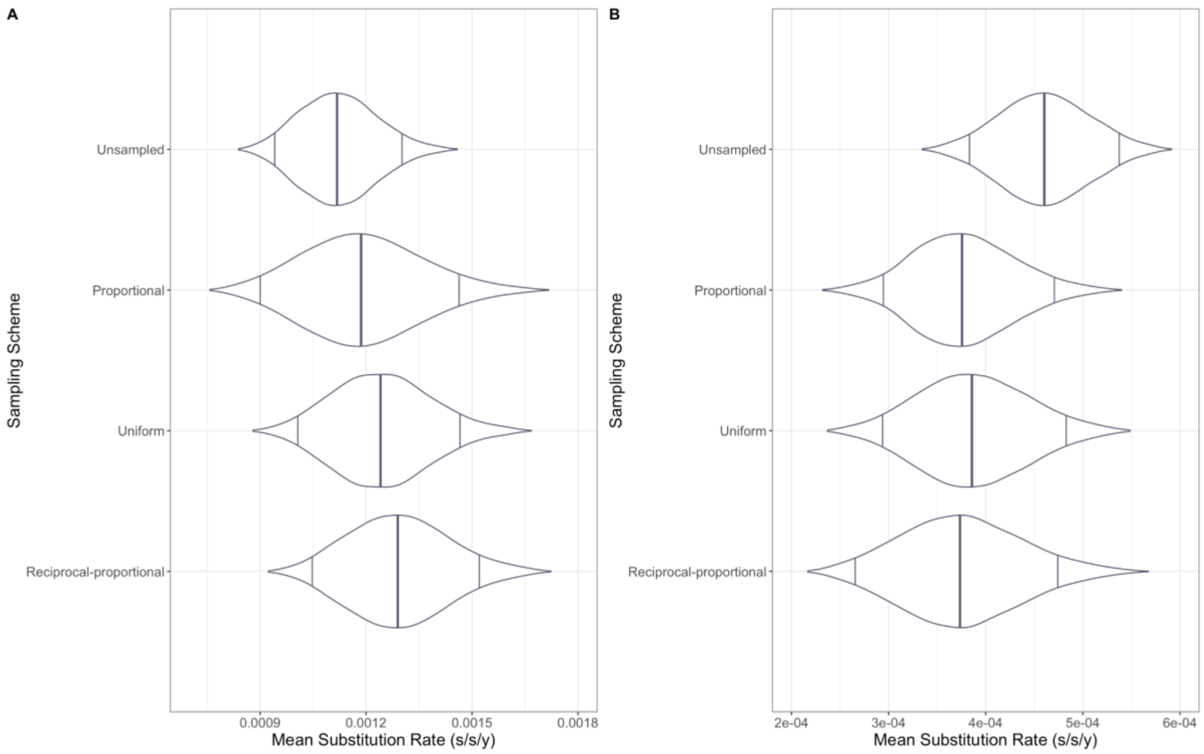

Supplementary Figure 5: Mean substitution rate (s/s/y) for Hong Kong and Brazil. Figure 1A represents Hong Kong with Figure 1B representing the Amazonas. The central line represents the posterior mean and with intervals representing 95% Highest Posterior Density Interval

Supplementary Table 2: Accession ID of each Hong Kong sequence for each sampling strategy used within this study

Unsampled	Proportional	Uniform	Reciprocal-proportional
EPI_ISL_ 412028	EPI_ISL_ 414517	EPI_ISL_ 412029	EPI_ISL_ 412028
EPI_ISL_ 412029	EPI_ISL_ 414519	EPI_ISL_ 414517	EPI_ISL_ 412029
EPI_ISL_ 412030	EPI_ISL_ 414527	EPI_ISL_ 414519	EPI_ISL_ 412030
EPI_ISL_ 414517	EPI_ISL_ 418815	EPI_ISL_ 414527	EPI_ISL_ 414517
EPI_ISL_ 414519	EPI_ISL_ 419224	EPI_ISL_ 414569	EPI_ISL_ 414519
EPI_ISL_ 414527	EPI_ISL_ 419229	EPI_ISL_ 414571	EPI_ISL_ 414527
EPI_ISL_ 414528	EPI_ISL_ 419232	EPI_ISL_ 416314	EPI_ISL_ 414528
EPI_ISL_ 414569	EPI_ISL_ 450404	EPI_ISL_ 417064	EPI_ISL_ 414569
EPI_ISL_ 414571	EPI_ISL_ 450405	EPI_ISL_ 417443	EPI_ISL_ 414571
EPI_ISL_ 416314	EPI_ISL_ 450410	EPI_ISL_ 419214	EPI_ISL_ 416314
EPI_ISL_ 417064	EPI_ISL_ 476801	EPI_ISL_ 419215	EPI_ISL_ 417064
EPI_ISL_ 417176	EPI_ISL_ 476802	EPI_ISL_ 419217	EPI_ISL_ 417176
EPI_ISL_ 417178	EPI_ISL_ 476803	EPI_ISL_ 419224	EPI_ISL_ 417178
EPI_ISL_ 417181	EPI_ISL_ 497769	EPI_ISL_ 419225	EPI_ISL_ 417181
EPI_ISL_ 417185	EPI_ISL_ 497773	EPI_ISL_ 419227	EPI_ISL_ 417185
EPI_ISL_ 417187	EPI_ISL_ 497775	EPI_ISL_ 419228	EPI_ISL_ 417187
EPI_ISL_ 417188	EPI_ISL_ 497784	EPI_ISL_ 419229	EPI_ISL_ 417188
EPI_ISL_ 417193	EPI_ISL_ 497786	EPI_ISL_ 419231	EPI_ISL_ 417193
EPI_ISL_ 417197	EPI_ISL_ 497791	EPI_ISL_ 419232	EPI_ISL_ 417197
EPI_ISL_ 417443	EPI_ISL_ 497796	EPI_ISL_ 419245	EPI_ISL_ 417443
EPI_ISL_ 418815	EPI_ISL_ 497799	EPI_ISL_ 419247	EPI_ISL_ 418815
EPI_ISL_ 419214	EPI_ISL_ 497806	EPI_ISL_ 419250	EPI_ISL_ 419214
EPI_ISL_ 419215	EPI_ISL_ 497808	EPI_ISL_ 419252	EPI_ISL_ 419215
EPI_ISL_ 419216	EPI_ISL_ 497810	EPI_ISL_ 434564	EPI_ISL_ 419216
EPI_ISL_ 419217	EPI_ISL_ 497811	EPI_ISL_ 434565	EPI_ISL_ 419217
EPI_ISL_ 419219	EPI_ISL_ 497818	EPI_ISL_ 434567	EPI_ISL_ 419219
EPI_ISL_ 419221	EPI_ISL_ 497819	EPI_ISL_ 434568	EPI_ISL_ 419221
EPI_ISL_ 419222	EPI_ISL_ 497821	EPI_ISL_ 434569	EPI_ISL_ 419222
EPI_ISL_ 419224	EPI_ISL_ 497823	EPI_ISL_ 434570	EPI_ISL_ 419224

EPI_ISL_ 419225	EPI_ISL_ 497824	EPI_ISL_ 434571	EPI_ISL_ 419225
EPI_ISL_ 419226	EPI_ISL_ 497840	EPI_ISL_ 450405	EPI_ISL_ 419226
EPI_ISL_ 419227	EPI_ISL_ 497845	EPI_ISL_ 450408	EPI_ISL_ 419227
EPI_ISL_ 419228	EPI_ISL_ 497846	EPI_ISL_ 450409	EPI_ISL_ 419228
EPI_ISL_ 419229	EPI_ISL_ 497847	EPI_ISL_ 450410	EPI_ISL_ 419229
EPI_ISL_ 419231	EPI_ISL_ 497850	EPI_ISL_ 450411	EPI_ISL_ 419231
EPI_ISL_ 419232	EPI_ISL_ 497856	EPI_ISL_ 476801	EPI_ISL_ 419232
EPI_ISL_ 419245	EPI_ISL_ 497865	EPI_ISL_ 476802	EPI_ISL_ 419245
EPI_ISL_ 419247	EPI_ISL_ 497870	EPI_ISL_ 476804	EPI_ISL_ 419247
EPI_ISL_ 419250	EPI_ISL_ 516798	EPI_ISL_ 497769	EPI_ISL_ 419250
EPI_ISL_ 419252	EPI_ISL_ 539820	EPI_ISL_ 497771	EPI_ISL_ 419252
EPI_ISL_ 434560	EPI_ISL_ 539850	EPI_ISL_ 497783	EPI_ISL_ 434563
EPI_ISL_ 434563	EPI_ISL_ 539851	EPI_ISL_ 497784	EPI_ISL_ 434564
EPI_ISL_ 434564	EPI_ISL_ 610167	EPI_ISL_ 497791	EPI_ISL_ 434565
EPI_ISL_ 434565	EPI_ISL_ 610168	EPI_ISL_ 497806	EPI_ISL_ 434566
EPI_ISL_ 434566	EPI_ISL_ 610169	EPI_ISL_ 497810	EPI_ISL_ 434567
EPI_ISL_ 434567	EPI_ISL_ 610170	EPI_ISL_ 497811	EPI_ISL_ 434568
EPI_ISL_ 434568	EPI_ISL_ 610171	EPI_ISL_ 497813	EPI_ISL_ 434569
EPI_ISL_ 434569	EPI_ISL_ 610172	EPI_ISL_ 497818	EPI_ISL_ 434570
EPI_ISL_ 434570	EPI_ISL_ 610173	EPI_ISL_ 497821	EPI_ISL_ 434571
EPI_ISL_ 434571	EPI_ISL_ 610174	EPI_ISL_ 497823	EPI_ISL_ 450405
EPI_ISL_ 450404	EPI_ISL_ 610175	EPI_ISL_ 497824	EPI_ISL_ 450408
EPI_ISL_ 450405	EPI_ISL_ 610177	EPI_ISL_ 497826	EPI_ISL_ 450409
EPI_ISL_ 450408		EPI_ISL_ 497827	EPI_ISL_ 450410
EPI_ISL_ 450409		EPI_ISL_ 497831	EPI_ISL_ 450411
EPI_ISL_ 450410		EPI_ISL_ 497832	EPI_ISL_ 450412
EPI_ISL_ 450411		EPI_ISL_ 497846	EPI_ISL_ 476802
EPI_ISL_ 450412		EPI_ISL_ 497847	EPI_ISL_ 476804
EPI_ISL_ 476801		EPI_ISL_ 497848	EPI_ISL_ 497769
EPI_ISL_ 476802		EPI_ISL_ 497856	EPI_ISL_ 497771
EPI_ISL_ 476803		EPI_ISL_ 497860	EPI_ISL_ 497773

EPI_ISL_ 476804		EPI_ISL_ 497865	EPI_ISL_ 497783
EPI_ISL_ 497769		EPI_ISL_ 539820	EPI_ISL_ 497784
EPI_ISL_ 497771		EPI_ISL_ 539850	EPI_ISL_ 497791
EPI_ISL_ 497773		EPI_ISL_ 539851	EPI_ISL_ 497797
EPI_ISL_ 497775		EPI_ISL_ 610165	EPI_ISL_ 497811
EPI_ISL_ 497783		EPI_ISL_ 610166	EPI_ISL_ 497812
EPI_ISL_ 497784		EPI_ISL_ 610167	EPI_ISL_ 497818
EPI_ISL_ 497786		EPI_ISL_ 610168	EPI_ISL_ 497819
EPI_ISL_ 497791		EPI_ISL_ 610169	EPI_ISL_ 497823
EPI_ISL_ 497796		EPI_ISL_ 610171	EPI_ISL_ 497824
EPI_ISL_ 497797		EPI_ISL_ 610173	EPI_ISL_ 497827
EPI_ISL_ 497798		EPI_ISL_ 610174	EPI_ISL_ 497831
EPI_ISL_ 497799		EPI_ISL_ 610175	EPI_ISL_ 497833
EPI_ISL_ 497806		EPI_ISL_ 610177	EPI_ISL_ 497848
EPI_ISL_ 497808			EPI_ISL_ 497850
EPI_ISL_ 497810			EPI_ISL_ 497856
EPI_ISL_ 497811			EPI_ISL_ 497860
EPI_ISL_ 497812			EPI_ISL_ 497864
EPI_ISL_ 497813			EPI_ISL_ 497865
EPI_ISL_ 497818			EPI_ISL_ 539850
EPI_ISL_ 497819			EPI_ISL_ 539851
EPI_ISL_ 497820			EPI_ISL_ 610165
EPI_ISL_ 497821			EPI_ISL_ 610166
EPI_ISL_ 497823			EPI_ISL_ 610172
EPI_ISL_ 497824			EPI_ISL_ 610177
EPI_ISL_ 497826			
EPI_ISL_ 497827			
EPI_ISL_ 497831			

EPI_ISL_ 497832			
EPI_ISL_ 497833			
EPI_ISL_ 497840			
EPI_ISL_ 497845			
EPI_ISL_ 497846			
EPI_ISL_ 497847			
EPI_ISL_ 497848			
EPI_ISL_ 497850			
EPI_ISL_ 497856			
EPI_ISL_ 497860			
EPI_ISL_ 497864			
EPI_ISL_ 497865			
EPI_ISL_ 497870			
EPI_ISL_ 516798			
EPI_ISL_ 539820			
EPI_ISL_ 539850			
EPI_ISL_ 539851			
EPI_ISL_ 610165			
EPI_ISL_ 610166			
EPI_ISL_ 610167			
EPI_ISL_ 610168			
EPI_ISL_ 610169			
EPI_ISL_ 610170			
EPI_ISL_ 610171			
EPI_ISL_ 610172			
EPI_ISL_ 610173			
EPI_ISL_ 610174			
EPI_ISL_ 610175			

EPI_ISL_ 610177			

Supplementary Table 3: Accession ID of each Amazonas State, Brazil sequence for each sampling strategy used within this study

Unsampled	Proportional	Uniform	Reciprocal-proportional
EPI_ISL_ 1034306	EPI_ISL_ 1034304	EPI_ISL_ 1034304	EPI_ISL_ 1034306
EPI_ISL_ 1060876	EPI_ISL_ 1034306	EPI_ISL_ 1034306	EPI_ISL_ 1060913
EPI_ISL_ 1060877	EPI_ISL_ 1060877	EPI_ISL_ 1060877	EPI_ISL_ 1060914
EPI_ISL_ 1060881	EPI_ISL_ 1060881	EPI_ISL_ 1060881	EPI_ISL_ 1068149
EPI_ISL_ 1060888	EPI_ISL_ 1060897	EPI_ISL_ 1060888	EPI_ISL_ 1068150
EPI_ISL_ 1060889	EPI_ISL_ 1060900	EPI_ISL_ 1060889	EPI_ISL_ 1068156
EPI_ISL_ 1060894	EPI_ISL_ 1060902	EPI_ISL_ 1060897	EPI_ISL_ 1068198
EPI_ISL_ 1060897	EPI_ISL_ 1060904	EPI_ISL_ 1060900	EPI_ISL_ 1068258
EPI_ISL_ 1060900	EPI_ISL_ 1060906	EPI_ISL_ 1060912	EPI_ISL_ 1068260
EPI_ISL_ 1060902	EPI_ISL_ 1060912	EPI_ISL_ 1060913	EPI_ISL_ 1068262
EPI_ISL_ 1060904	EPI_ISL_ 1060913	EPI_ISL_ 1060956	EPI_ISL_ 1068263
EPI_ISL_ 1060906	EPI_ISL_ 1060914	EPI_ISL_ 1061026	EPI_ISL_ 1068264
EPI_ISL_ 1060911	EPI_ISL_ 1060918	EPI_ISL_ 1068111	EPI_ISL_ 1068278
EPI_ISL_ 1060912	EPI_ISL_ 1060956	EPI_ISL_ 1068149	EPI_ISL_ 1068286
EPI_ISL_ 1060913	EPI_ISL_ 1061026	EPI_ISL_ 1068150	EPI_ISL_ 1068288
EPI_ISL_ 1060914	EPI_ISL_ 1068110	EPI_ISL_ 1068154	EPI_ISL_ 1166615
EPI_ISL_ 1060918	EPI_ISL_ 1068111	EPI_ISL_ 1068158	EPI_ISL_ 1213190
EPI_ISL_ 1060956	EPI_ISL_ 1068112	EPI_ISL_ 1068160	EPI_ISL_ 1261690
EPI_ISL_ 1061026	EPI_ISL_ 1068114	EPI_ISL_ 1068169	EPI_ISL_ 1261694
EPI_ISL_ 1068110	EPI_ISL_ 1068149	EPI_ISL_ 1068198	EPI_ISL_ 2777236
EPI_ISL_ 1068111	EPI_ISL_ 1068150	EPI_ISL_ 1068222	EPI_ISL_ 2777320
EPI_ISL_ 1068112	EPI_ISL_ 1068151	EPI_ISL_ 1068225	EPI_ISL_ 2777363
EPI_ISL_ 1068114	EPI_ISL_ 1068154	EPI_ISL_ 1068226	EPI_ISL_ 2777375
EPI_ISL_ 1068149	EPI_ISL_ 1068156	EPI_ISL_ 1068243	EPI_ISL_ 2777376
EPI_ISL_ 1068150	EPI_ISL_ 1068158	EPI_ISL_ 1068248	EPI_ISL_ 2777384
EPI_ISL_ 1068151	EPI_ISL_ 1068160	EPI_ISL_ 1068249	EPI_ISL_ 2777388
EPI_ISL_ 1068154	EPI_ISL_ 1068169	EPI_ISL_ 1068260	EPI_ISL_ 2777397

EPI_ISL_ 1068156	EPI_ISL_ 1068198	EPI_ISL_ 1068261	EPI_ISL_ 2777399
EPI_ISL_ 1068158	EPI_ISL_ 1068221	EPI_ISL_ 1068262	EPI_ISL_ 2777401
EPI_ISL_ 1068160	EPI_ISL_ 1068222	EPI_ISL_ 1068263	EPI_ISL_ 2777403
EPI_ISL_ 1068169	EPI_ISL_ 1068225	EPI_ISL_ 1068264	EPI_ISL_ 2777404
EPI_ISL_ 1068198	EPI_ISL_ 1068248	EPI_ISL_ 1068266	EPI_ISL_ 2777409
EPI_ISL_ 1068221	EPI_ISL_ 1068249	EPI_ISL_ 1068268	EPI_ISL_ 2777410
EPI_ISL_ 1068222	EPI_ISL_ 1068258	EPI_ISL_ 1068269	EPI_ISL_ 2777414
EPI_ISL_ 1068225	EPI_ISL_ 1068260	EPI_ISL_ 1068270	EPI_ISL_ 2777415
EPI_ISL_ 1068226	EPI_ISL_ 1068261	EPI_ISL_ 1068271	EPI_ISL_ 2777465
EPI_ISL_ 1068243	EPI_ISL_ 1068262	EPI_ISL_ 1068272	EPI_ISL_ 2777466
EPI_ISL_ 1068248	EPI_ISL_ 1068263	EPI_ISL_ 1068273	EPI_ISL_ 2777467
EPI_ISL_ 1068249	EPI_ISL_ 1068264	EPI_ISL_ 1068274	EPI_ISL_ 2777469
EPI_ISL_ 1068258	EPI_ISL_ 1068266	EPI_ISL_ 1068279	EPI_ISL_ 2777470
EPI_ISL_ 1068260	EPI_ISL_ 1068268	EPI_ISL_ 1068282	EPI_ISL_ 2777472
EPI_ISL_ 1068261	EPI_ISL_ 1068269	EPI_ISL_ 1068283	EPI_ISL_ 2777473
EPI_ISL_ 1068262	EPI_ISL_ 1068270	EPI_ISL_ 1068284	EPI_ISL_ 2777474
EPI_ISL_ 1068263	EPI_ISL_ 1068271	EPI_ISL_ 1068285	EPI_ISL_ 2777475
EPI_ISL_ 1068264	EPI_ISL_ 1068272	EPI_ISL_ 1068286	EPI_ISL_ 2777482
EPI_ISL_ 1068266	EPI_ISL_ 1068273	EPI_ISL_ 1068287	EPI_ISL_ 2777483
EPI_ISL_ 1068268	EPI_ISL_ 1068274	EPI_ISL_ 1068288	EPI_ISL_ 2777485
EPI_ISL_ 1068269	EPI_ISL_ 1068275	EPI_ISL_ 1068290	EPI_ISL_ 2777503
EPI_ISL_ 1068270	EPI_ISL_ 1068276	EPI_ISL_ 1068291	EPI_ISL_ 2777508
EPI_ISL_ 1068271	EPI_ISL_ 1068278	EPI_ISL_ 1068292	EPI_ISL_ 2777509
EPI_ISL_ 1068272	EPI_ISL_ 1068279	EPI_ISL_ 1166615	EPI_ISL_ 2777516
EPI_ISL_ 1068273	EPI_ISL_ 1068280	EPI_ISL_ 1213190	EPI_ISL_ 2777599
EPI_ISL_ 1068274	EPI_ISL_ 1068281	EPI_ISL_ 1213204	EPI_ISL_ 2777698
EPI_ISL_ 1068275	EPI_ISL_ 1068282	EPI_ISL_ 1261683	EPI_ISL_ 2777986
EPI_ISL_ 1068276	EPI_ISL_ 1068283	EPI_ISL_ 1261685	EPI_ISL_ 2777987
EPI_ISL_ 1068278	EPI_ISL_ 1068284	EPI_ISL_ 1261690	EPI_ISL_ 2777993
EPI_ISL_ 1068279	EPI_ISL_ 1068285	EPI_ISL_ 1261694	EPI_ISL_ 2777999
EPI_ISL_ 1068280	EPI_ISL_ 1068286	EPI_ISL_ 2777236	EPI_ISL_ 2778002
EPI_ISL_ 1068281	EPI_ISL_ 1068287	EPI_ISL_ 2777248	EPI_ISL_ 2778004
EPI_ISL_ 1068282	EPI_ISL_ 1068288	EPI_ISL_ 2777249	EPI_ISL_ 2778005

EPI_ISL_ 1068283	EPI_ISL_ 1068289	EPI_ISL_ 2777250	EPI_ISL_ 833138
EPI_ISL_ 1068284	EPI_ISL_ 1068290	EPI_ISL_ 2777320	EPI_ISL_ 833140
EPI_ISL_ 1068285	EPI_ISL_ 1068291	EPI_ISL_ 2777363	EPI_ISL_ 906071
EPI_ISL_ 1068286	EPI_ISL_ 1068292	EPI_ISL_ 2777364	EPI_ISL_ 918505
EPI_ISL_ 1068287	EPI_ISL_ 1166615	EPI_ISL_ 2777373	EPI_ISL_ 918506
EPI_ISL_ 1068288	EPI_ISL_ 1213190	EPI_ISL_ 2777374	EPI_ISL_ 918508
EPI_ISL_ 1068289	EPI_ISL_ 1213204	EPI_ISL_ 2777375	EPI_ISL_ 918509
EPI_ISL_ 1068290	EPI_ISL_ 1261683	EPI_ISL_ 2777376	
EPI_ISL_ 1068291	EPI_ISL_ 1261685	EPI_ISL_ 2777377	
EPI_ISL_ 1068292	EPI_ISL_ 1261690	EPI_ISL_ 2777378	
EPI_ISL_ 1166615	EPI_ISL_ 1261694	EPI_ISL_ 2777380	
EPI_ISL_ 1213190	EPI_ISL_ 2777236	EPI_ISL_ 2777383	
EPI_ISL_ 1213204	EPI_ISL_ 2777238	EPI_ISL_ 2777384	
EPI_ISL_ 1261683	EPI_ISL_ 2777248	EPI_ISL_ 2777385	
EPI_ISL_ 1261685	EPI_ISL_ 2777249	EPI_ISL_ 2777388	
EPI_ISL_ 1261690	EPI_ISL_ 2777250	EPI_ISL_ 2777397	
EPI_ISL_ 1261694	EPI_ISL_ 2777251	EPI_ISL_ 2777398	
EPI_ISL_ 2777236	EPI_ISL_ 2777320	EPI_ISL_ 2777399	
EPI_ISL_ 2777238	EPI_ISL_ 2777363	EPI_ISL_ 2777400	
EPI_ISL_ 2777248	EPI_ISL_ 2777364	EPI_ISL_ 2777401	
EPI_ISL_ 2777249	EPI_ISL_ 2777373	EPI_ISL_ 2777402	
EPI_ISL_ 2777250	EPI_ISL_ 2777374	EPI_ISL_ 2777403	
EPI_ISL_ 2777251	EPI_ISL_ 2777375	EPI_ISL_ 2777404	
EPI_ISL_ 2777320	EPI_ISL_ 2777376	EPI_ISL_ 2777405	
EPI_ISL_ 2777363	EPI_ISL_ 2777377	EPI_ISL_ 2777406	
EPI_ISL_ 2777364	EPI_ISL_ 2777378	EPI_ISL_ 2777407	
EPI_ISL_ 2777373	EPI_ISL_ 2777380	EPI_ISL_ 2777408	
EPI_ISL_ 2777374	EPI_ISL_ 2777382	EPI_ISL_ 2777410	
EPI_ISL_ 2777375	EPI_ISL_ 2777383	EPI_ISL_ 2777412	
EPI_ISL_ 2777376	EPI_ISL_ 2777384	EPI_ISL_ 2777413	
EPI_ISL_ 2777377	EPI_ISL_ 2777385	EPI_ISL_ 2777414	
EPI_ISL_ 2777378	EPI_ISL_ 2777388	EPI_ISL_ 2777415	
EPI_ISL_ 2777380	EPI_ISL_ 2777397	EPI_ISL_ 2777417	

EPI_ISL_ 2777382	EPI_ISL_ 2777398	EPI_ISL_ 2777418	
EPI_ISL_ 2777383	EPI_ISL_ 2777399	EPI_ISL_ 2777419	
EPI_ISL_ 2777384	EPI_ISL_ 2777400	EPI_ISL_ 2777454	
EPI_ISL_ 2777385	EPI_ISL_ 2777401	EPI_ISL_ 2777461	
EPI_ISL_ 2777388	EPI_ISL_ 2777402	EPI_ISL_ 2777462	
EPI_ISL_ 2777397	EPI_ISL_ 2777403	EPI_ISL_ 2777465	
EPI_ISL_ 2777398	EPI_ISL_ 2777404	EPI_ISL_ 2777466	
EPI_ISL_ 2777399	EPI_ISL_ 2777405	EPI_ISL_ 2777467	
EPI_ISL_ 2777400	EPI_ISL_ 2777406	EPI_ISL_ 2777469	
EPI_ISL_ 2777401	EPI_ISL_ 2777407	EPI_ISL_ 2777470	
EPI_ISL_ 2777402	EPI_ISL_ 2777408	EPI_ISL_ 2777472	
EPI_ISL_ 2777403	EPI_ISL_ 2777409	EPI_ISL_ 2777473	
EPI_ISL_ 2777404	EPI_ISL_ 2777410	EPI_ISL_ 2777474	
EPI_ISL_ 2777405	EPI_ISL_ 2777412	EPI_ISL_ 2777475	
EPI_ISL_ 2777406	EPI_ISL_ 2777413	EPI_ISL_ 2777477	
EPI_ISL_ 2777407	EPI_ISL_ 2777414	EPI_ISL_ 2777478	
EPI_ISL_ 2777408	EPI_ISL_ 2777415	EPI_ISL_ 2777479	
EPI_ISL_ 2777409	EPI_ISL_ 2777416	EPI_ISL_ 2777481	
EPI_ISL_ 2777410	EPI_ISL_ 2777417	EPI_ISL_ 2777482	
EPI_ISL_ 2777412	EPI_ISL_ 2777418	EPI_ISL_ 2777483	
EPI_ISL_ 2777413	EPI_ISL_ 2777419	EPI_ISL_ 2777485	
EPI_ISL_ 2777414	EPI_ISL_ 2777420	EPI_ISL_ 2777495	
EPI_ISL_ 2777415	EPI_ISL_ 2777454	EPI_ISL_ 2777498	
EPI_ISL_ 2777416	EPI_ISL_ 2777460	EPI_ISL_ 2777503	
EPI_ISL_ 2777417	EPI_ISL_ 2777461	EPI_ISL_ 2777507	
EPI_ISL_ 2777418	EPI_ISL_ 2777462	EPI_ISL_ 2777508	
EPI_ISL_ 2777419	EPI_ISL_ 2777464	EPI_ISL_ 2777539	
EPI_ISL_ 2777420	EPI_ISL_ 2777466	EPI_ISL_ 2777599	
EPI_ISL_ 2777454	EPI_ISL_ 2777467	EPI_ISL_ 2777698	
EPI_ISL_ 2777460	EPI_ISL_ 2777468	EPI_ISL_ 2777700	
EPI_ISL_ 2777461	EPI_ISL_ 2777469	EPI_ISL_ 2777701	
EPI_ISL_ 2777462	EPI_ISL_ 2777470	EPI_ISL_ 2777740	
EPI_ISL_ 2777464	EPI_ISL_ 2777472	EPI_ISL_ 2777986	

EPI_ISL_ 2777465	EPI_ISL_ 2777473	EPI_ISL_ 2777987	
EPI_ISL_ 2777466	EPI_ISL_ 2777475	EPI_ISL_ 2777993	
EPI_ISL_ 2777467	EPI_ISL_ 2777477	EPI_ISL_ 2777995	
EPI_ISL_ 2777468	EPI_ISL_ 2777478	EPI_ISL_ 2777996	
EPI_ISL_ 2777469	EPI_ISL_ 2777481	EPI_ISL_ 2777997	
EPI_ISL_ 2777470	EPI_ISL_ 2777482	EPI_ISL_ 2777998	
EPI_ISL_ 2777471	EPI_ISL_ 2777495	EPI_ISL_ 2777999	
EPI_ISL_ 2777472	EPI_ISL_ 2777498	EPI_ISL_ 2778000	
EPI_ISL_ 2777473	EPI_ISL_ 2777499	EPI_ISL_ 2778002	
EPI_ISL_ 2777474	EPI_ISL_ 2777503	EPI_ISL_ 2778005	
EPI_ISL_ 2777475	EPI_ISL_ 2777508	EPI_ISL_ 811149	
EPI_ISL_ 2777477	EPI_ISL_ 2777516	EPI_ISL_ 833136	
EPI_ISL_ 2777478	EPI_ISL_ 2777539	EPI_ISL_ 833139	
EPI_ISL_ 2777479	EPI_ISL_ 2777698	EPI_ISL_ 833140	
EPI_ISL_ 2777481	EPI_ISL_ 2777701	EPI_ISL_ 906071	
EPI_ISL_ 2777482	EPI_ISL_ 2777740	EPI_ISL_ 906077	
EPI_ISL_ 2777483	EPI_ISL_ 2777986	EPI_ISL_ 906081	
EPI_ISL_ 2777484	EPI_ISL_ 2777987	EPI_ISL_ 918500	
EPI_ISL_ 2777485	EPI_ISL_ 2777995	EPI_ISL_ 918502	
EPI_ISL_ 2777495	EPI_ISL_ 2777996	EPI_ISL_ 918503	
EPI_ISL_ 2777498	EPI_ISL_ 2777997	EPI_ISL_ 918506	
EPI_ISL_ 2777499	EPI_ISL_ 2777998	EPI_ISL_ 918508	
EPI_ISL_ 2777503	EPI_ISL_ 2778002	EPI_ISL_ 918509	
EPI_ISL_ 2777507	EPI_ISL_ 2778005	EPI_ISL_ 918511	
EPI_ISL_ 2777508	EPI_ISL_ 811149		
EPI_ISL_ 2777509	EPI_ISL_ 833136		
EPI_ISL_ 2777516	EPI_ISL_ 833138		
EPI_ISL_ 2777539	EPI_ISL_ 833139		
EPI_ISL_ 2777599	EPI_ISL_ 833140		
EPI_ISL_ 2777698	EPI_ISL_ 906071		
EPI_ISL_ 2777700	EPI_ISL_ 906080		
EPI_ISL_ 2777701	EPI_ISL_ 906081		
EPI_ISL_ 2777740	EPI_ISL_ 918500		

EPI_ISL_ 2777986	EPI_ISL_ 918501		
EPI_ISL_ 2777987	EPI_ISL_ 918502		
EPI_ISL_ 2777993	EPI_ISL_ 918503		
EPI_ISL_ 2777995	EPI_ISL_ 918505		
EPI_ISL_ 2777996	EPI_ISL_ 918506		
EPI_ISL_ 2777997	EPI_ISL_ 918507		
EPI_ISL_ 2777998	EPI_ISL_ 918508		
EPI_ISL_ 2777999	EPI_ISL_ 918510		
EPI_ISL_ 2778000	EPI_ISL_ 918511		
EPI_ISL_ 2778002			
EPI_ISL_ 2778004			
EPI_ISL_ 2778005			
EPI_ISL_ 811149			
EPI_ISL_ 833136			
EPI_ISL_ 833138			
EPI_ISL_ 833139			
EPI_ISL_ 833140			
EPI_ISL_ 906071			
EPI_ISL_ 906075			
EPI_ISL_ 906076			
EPI_ISL_ 906077			
EPI_ISL_ 906080			
EPI_ISL_ 906081			
EPI_ISL_ 918499			
EPI_ISL_ 918500			
EPI_ISL_ 918501			
EPI_ISL_ 918502			
EPI_ISL_ 918503			
EPI_ISL_ 918504			
EPI_ISL_ 918505			
EPI_ISL_ 918506			
EPI_ISL_ 918507			
EPI_ISL_ 918508			

EPI_ISL_ 918509			
EPI_ISL_ 918510			
EPI_ISL_ 918511			

Supplementary Table 4: GISAID acknowledgement table

We gratefully acknowledge the following Authors from the Originating laboratories responsible for obtaining the specimens, as well as the Submitting laboratories where the genome data were generated and shared via GISAID, on which this research is based.

All Submitters of data may be contacted directly via www.gisaid.org

Authors are sorted alphabetically.

Acknowledgement EPI_SET Identifier: EPI_SET_220804pn

Accession ID	Originating Laboratory	Submitting Laboratory	Authors
EPI_ISL_1060877, EPI_ISL_1060881, EPI_ISL_1060888, EPI_ISL_1060889, EPI_ISL_1060894, EPI_ISL_1060897, EPI_ISL_1060906, EPI_ISL_1060911, EPI_ISL_1060912, EPI_ISL_1060913, EPI_ISL_1060956, EPI_ISL_1061026			
see above	CDL Laboratorio Santos e Vidal LTDA.	Instituto de Medicina Tropical de Sao Paulo	Brazil-UK Centre for Arbovirus Discovery Diagnosis Genomics and Epidemiology (CADDE) Genomic Network - Instituto de Medicina Tropical
EPI_ISL_1060876, EPI_ISL_1060900, EPI_ISL_1060902, EPI_ISL_1060904, EPI_ISL_1060914, EPI_ISL_1060918	DB Diagnosticos do Brasil	Instituto de Medicina Tropical de Sao Paulo	Brazil-UK Centre for Arbovirus Discovery Diagnosis Genomics and Epidemiology (CADDE) Genomic Network - Instituto de Medicina Tropical
EPI_ISL_417187, EPI_ISL_417188, EPI_ISL_417193, EPI_ISL_417197, EPI_ISL_418815, EPI_ISL_419214, EPI_ISL_419215, EPI_ISL_419216, EPI_ISL_419219, EPI_ISL_419224, EPI_ISL_419225, EPI_ISL_419226, EPI_ISL_419227, EPI_ISL_419228, EPI_ISL_419229, EPI_ISL_419232, EPI_ISL_419245, EPI_ISL_419247, EPI_ISL_419250, EPI_ISL_419252			
see above	Department of Clinical Pathology, Pamela Youde Nethersole Eastern Hospital	Department of Health Technology and Informatics, Faculty of Health and Social Science, The Hong Kong Polytechnic University	Alan Ka-Lun WU; Alex Yat-Man HO; Barry Kin-Chung WONG; David Ho-Keung SHUM; Eugene Yuk-Keung TSO; Gilman Kit-Hang SIU; Hiu-Yin LAO; Kam-Tong YIP; Kam-Tong Yip; Kenneth Siu-Sing LEUNG; Kingsley King-Gee TAM; Kit-Man SIN; Kitty Sau-Chun FUNG; Kwok-Cheung LUNG; Lam-Kwong LEE; Man-Chun CHAN; Ming-Pan CHOI; Miranda Chong-Yee YAU; Raymond Wai-To LIU; Sandy Ka-Yee CHAU; Shea Ping YIP; Tak-Lun QUE; Tak-Lun Que; Timothy Ting-Leung NG; Wai-Shing LEUNG; Wing Cheong YAM; Wing-Kin TO; Yuk-Yung NG
EPI_ISL_419231	Department of Clinical Pathology, Tuen Mun Hospital, 23 Tsing Chung Koon Road, Tuen Mun, N.T.	Department of Health Technology and Informatics, Faculty of Health and Social Science, The Hong Kong Polytechnic University	Alan Ka-Lun WU; Alex Yat-Man HO; Barry Kin-Chung WONG; David Ho-Keung SHUM; Eugene Yuk-Keung TSO; Gilman Kit-Hang SIU; Hiu-Yin LAO; Kam-Tong YIP; Kenneth Siu-Sing LEUNG; Kingsley King-Gee TAM; Kit-Man SIN; Kitty Sau-Chun FUNG; Kwok-Cheung LUNG; Lam-Kwong LEE; Man-Chun CHAN; Ming-Pan CHOI; Miranda Chong-Yee YAU; Raymond Wai-To LIU; Sandy Ka-Yee CHAU; Shea Ping YIP; Tak-Lun QUE; Timothy Ting-Leung NG; Wai-Shing LEUNG; Wing Cheong YAM; Wing-Kin TO; Yuk-Yung NG
EPI_ISL_610165, EPI_ISL_610166, EPI_ISL_610167, EPI_ISL_610168, EPI_ISL_610169, EPI_ISL_610170, EPI_ISL_610171, EPI_ISL_610172, EPI_ISL_610173, EPI_ISL_610174, EPI_ISL_610175, EPI_ISL_610177			
see above	Department of Health Technology and Informatics, The Hong Kong Polytechnic University	Department of Health Technology and Informatics, The Hong Kong Polytechnic University	A.K.-L.; A.Y.-M.; B.K.-C.; C.T.-M.; Chan; Chau; D.H.-K.; Fung; G.K.-H.; H.-Y.; Ho; J.S.-L.; K.-T.; K.K.-G.; K.S.-C.; K.S.-S.; L.-K.; Lai; Lao; Lee; Leung; Luk, K.; M.C.-Y.; Ng; Que; S.K.-Y.; S.P.; Shum; Siu; T.-L.; T.T.-L.; Tam; To; W.-K.; W.C.; Wong; Wu; Y.W.-M.; Yam; Yau; Yip
EPI_ISL_416314	Department of Microbiology, Faculty of Medicine, The Chinese University of Hong Kong, Hong Kong SAR, China	Department of Microbiology, Faculty of Medicine, Chinese University of Hong Kong, Hong Kong SAR, China	Paul KS Chan; Zigui Chen
EPI_ISL_434560, EPI_ISL_497769, EPI_ISL_497771, EPI_ISL_497773, EPI_ISL_497775, EPI_ISL_497783, EPI_ISL_497784, EPI_ISL_497786, EPI_ISL_497791, EPI_ISL_497796, EPI_ISL_497797, EPI_ISL_497798, EPI_ISL_497799, EPI_ISL_497806, EPI_ISL_497808, EPI_ISL_497810, EPI_ISL_497811, EPI_ISL_497812, EPI_ISL_497813, EPI_ISL_497818, EPI_ISL_497819, EPI_ISL_497820, EPI_ISL_497821, EPI_ISL_497823, EPI_ISL_497824, EPI_ISL_497826, EPI_ISL_497827, EPI_ISL_497831, EPI_ISL_497832, EPI_ISL_497833, EPI_ISL_497840, EPI_ISL_497845, EPI_ISL_497846, EPI_ISL_497847, EPI_ISL_497848, EPI_ISL_497850, EPI_ISL_497856, EPI_ISL_497860, EPI_ISL_497864, EPI_ISL_497865, EPI_ISL_497870, EPI_ISL_516798			
see above	Department of Microbiology, The University of Hong Kong	Department of Microbiology, The University of Hong Kong	A.C.P.; Chan; Fung; Fung, J.; H.K.H.; He, Z.; K.S.C. and Woo; K.S.M.; Kelvin K.W. To; Kwok-Yung Yuen; Lau; Li; Luk; P.C.Y.; S.K.P.; T.T.Y.; Wong; Zhu, L.
EPI_ISL_417176, EPI_ISL_417178, EPI_ISL_419217, EPI_ISL_419222	Department of Pathology, Princess Margaret Hospital	Department of Health Technology and Informatics, Faculty of Health and Social Science, The Hong Kong Polytechnic University	Alan Ka-Lun WU; Alex Yat-Man HO; Barry Kin-Chung WONG; David Ho-Keung SHUM; Eugene Yuk-Keung TSO; Gilman Kit-Hang SIU; Hiu-Yin LAO; Kam-Tong YIP; Kam-Tong Yip; Kenneth Siu-Sing LEUNG; Kingsley King-Gee TAM; Kit-Man SIN; Kitty Sau-Chun FUNG; Kwok-Cheung LUNG; Lam-Kwong LEE; Man-Chun CHAN; Ming-Pan CHOI; Miranda Chong-Yee YAU; Raymond Wai-To LIU; Sandy Ka-Yee CHAU; Shea Ping YIP; Tak-Lun QUE; Tak-Lun Que; Timothy Ting-Leung NG; Wai-Shing LEUNG; Wing Cheong YAM; Wing-Kin TO; Yuk-Yung NG
EPI_ISL_417181, EPI_ISL_417185, EPI_ISL_419221	Department of Pathology, United Christian Hospital	Department of Health Technology and Informatics, Faculty of Health and Social Science, The Hong Kong Polytechnic University	Alan Ka-Lun WU; Alex Yat-Man HO; Barry Kin-Chung WONG; David Ho-Keung SHUM; Eugene Yuk-Keung TSO; Gilman Kit-Hang SIU; Hiu-Yin LAO; Kam-Tong YIP; Kam-Tong Yip; Kenneth Siu-Sing LEUNG; Kingsley King-Gee TAM; Kit-Man SIN; Kitty Sau-Chun FUNG; Kwok-Cheung LUNG; Lam-Kwong LEE; Man-Chun CHAN; Ming-Pan CHOI; Miranda Chong-Yee YAU; Raymond Wai-To LIU; Sandy Ka-Yee CHAU; Shea Ping YIP; Tak-Lun QUE; Tak-Lun Que; Timothy Ting-Leung NG; Wai-Shing LEUNG; Wing Cheong YAM; Wing-Kin TO; Yuk-Yung NG
EPI_ISL_412028, EPI_ISL_412030	Hong Kong Department of Health	School of Public Health, The University of Hon g Kong	Daniel K.W. Chu; Dominic N.C. Tsang; Leo L.M. Poon; Malik Peiris
EPI_ISL_414517, EPI_ISL_414519, EPI_ISL_414527, EPI_ISL_414528, EPI_ISL_414569, EPI_ISL_414571, EPI_ISL_476801, EPI_ISL_476802, EPI_ISL_476803, EPI_ISL_476804			
see above	Hong Kong Department of Health	School of Public Health, The University of Hong Kong	Daniel K.W. Chu; Dominic N.C. Tsang; Leo L.M. Poon; Malik Peiris
EPI_ISL_412029	Hong Kong Department of Health	The University of Hong Kong	Daniel K.W. Chu; Dominic N.C. Tsang; Leo L.M. Poon; Malik Peiris
EPI_ISL_906080, EPI_ISL_906081	Hospital Beneficiencia Portuguesa	Instituto Adolfo Lutz, Interdiciplinary Procedures Center, Strategic Laboratory	Claudia Regina Gonçalves; Claudio Tavares Sacchi; Erica Valessa Ramos Gomes; Karoline Rodrigues Campos
EPI_ISL_906075	Hospital Geral de Vila Penteado Dr Jose Pangella Sao Paulo	Instituto Adolfo Lutz, Interdiciplinary Procedures Center, Strategic Laboratory	Claudia Regina Gonçalves; Claudio Tavares Sacchi; Erica Valessa Ramos Gomes; Karoline Rodrigues Campos
EPI_ISL_906076, EPI_ISL_906077	Hospital Sao Luiz Sao Caetano	Instituto Adolfo Lutz, Interdiciplinary Procedures Center, Strategic Laboratory	Claudia Regina Gonçalves; Claudio Tavares Sacchi; Erica Valessa Ramos Gomes; Karoline Rodrigues Campos
EPI_ISL_918499, EPI_ISL_918500, EPI_ISL_918501, EPI_ISL_918502, EPI_ISL_918503, EPI_ISL_918504, EPI_ISL_918505, EPI_ISL_918506, EPI_ISL_918507, EPI_ISL_918508, EPI_ISL_918509, EPI_ISL_918510, EPI_ISL_918511, EPI_ISL_1261683, EPI_ISL_1261685, EPI_ISL_1261690, EPI_ISL_1261694			
see above	LACEN - Laboratório Central de Saúde Pública do Amazonas	Evandro Chagas Institute	A.M.; Barbagelata; E.C.; E.M.A.; Ferreira; J.A.; Junior; K.C.; L.C.; L.S.; M.C.; P.S.; Pinheiro; Santos; Silva; Sousa; Sousa Junior; W.D.C.; da Silva
EPI_ISL_1166615	LACEN - Laboratório Central de Saúde Pública do Rio Grande do Norte	Evandro Chagas Institute Virology	A.M.; Barbagelata; E.C.; E.M.A.; Ferreira; J.A.; Junior; K.C.; L.C.; L.S.; M.C.; P.S.; Pinheiro; Santos; Silva; Sousa; Sousa Junior; W.D.C.; da Silva
EPI_ISL_906071	LACEN-PI DR. Costa Alvarenga	Instituto Adolfo Lutz, Interdiciplinary Procedures Center, Strategic Laboratory	Claudia Regina Gonçalves; Claudio Tavares Sacchi; Erica Valessa Ramos Gomes; Karoline Rodrigues Campos
EPI_ISL_1213190, EPI_ISL_1213204	LBM/UFPB	Bioinformatics Laboratory / LNCC	Alessandra P Lamarca; Alexandra L Gerber; Ana Paula Melo Mariano; Ana Paula de C Guimarães; Ana Tereza R Vasconcelos; Angela Maria Guimarães Santos; Bianca Mendes Maciel; Danielle Angst Secco; Eduardo Sérgio Soares Sousa; Eloiza Helena Campana; Francisco Paulo Freire Neto; George Rego Albuquerque; Kátia Castanho Scortecci; Lucymara Fassarella Agnez Lima; Luiz G P de Almeida; Luís Cristóvão Porto; Otavio J. Brustolini; Paulo Ricardo Nascimento; Ronaldo da Silva Francisco Jr; Sandra Rocha Gadelha; Selma Maria Bezerra Jeronimo; Vinícius Pietta Perez
EPI_ISL_811149, EPI_ISL_833136, EPI_ISL_833138, EPI_ISL_833139, EPI_ISL_833140, EPI_ISL_1034306, EPI_ISL_1068110, EPI_ISL_1068111, EPI_ISL_1068112, EPI_ISL_1068114, EPI_ISL_1068149, EPI_ISL_1068150, EPI_ISL_1068151, EPI_ISL_1068154, EPI_ISL_1068156, EPI_ISL_1068158, EPI_ISL_1068160, EPI_ISL_1068169, EPI_ISL_1068198, EPI_ISL_1068221, EPI_ISL_1068222, EPI_ISL_1068225, EPI_ISL_1068226, EPI_ISL_1068243, EPI_ISL_1068248, EPI_ISL_1068249, EPI_ISL_1068258, EPI_ISL_1068260, EPI_ISL_1068261, EPI_ISL_1068262, EPI_ISL_1068263, EPI_ISL_1068264, EPI_ISL_1068266, EPI_ISL_1068268, EPI_ISL_1068269, EPI_ISL_1068270, EPI_ISL_1068271, EPI_ISL_1068272, EPI_ISL_1068273, EPI_ISL_1068274, EPI_ISL_1068275, EPI_ISL_1068276, EPI_ISL_1068278, EPI_ISL_1068279, EPI_ISL_1068280, EPI_ISL_1068281, EPI_ISL_1068282, EPI_ISL_1068283, EPI_ISL_1068284, EPI_ISL_1068285, EPI_ISL_1068286, EPI_ISL_1068287, EPI_ISL_1068288, EPI_ISL_1068289, EPI_ISL_1068290, EPI_ISL_1068291, EPI_ISL_1068292, EPI_ISL_2777363, EPI_ISL_2777364, EPI_ISL_2777373, EPI_ISL_2777374, EPI_ISL_2777375, EPI_ISL_2777376, EPI_ISL_2777377, EPI_ISL_2777378, EPI_ISL_2777380, EPI_ISL_2777382, EPI_ISL_2777383, EPI_ISL_2777384, EPI_ISL_2777385, EPI_ISL_2777388, EPI_ISL_2777397, EPI_ISL_2777398, EPI_ISL_2777399, EPI_ISL_2777400, EPI_ISL_2777401, EPI_ISL_2777402, EPI_ISL_2777403, EPI_ISL_2777404, EPI_ISL_2777405, EPI_ISL_2777406, EPI_ISL_2777407, EPI_ISL_2777408, EPI_ISL_2777409, EPI_ISL_2777410, EPI_ISL_2777412, EPI_ISL_2777413, EPI_ISL_2777414, EPI_ISL_2777415, EPI_ISL_2777416, EPI_ISL_2777417, EPI_ISL_2777418, EPI_ISL_2777419, EPI_ISL_2777420, EPI_ISL_2777454, EPI_ISL_2777460, EPI_ISL_2777461, EPI_ISL_2777462, EPI_ISL_2777464, EPI_ISL_2777465, EPI_ISL_2777466, EPI_ISL_2777467, EPI_ISL_2777468, EPI_ISL_2777469, EPI_ISL_2777470, EPI_ISL_2777471, EPI_ISL_2777472, EPI_ISL_2777473, EPI_ISL_2777474, EPI_ISL_2777475, EPI_ISL_2777477, EPI_ISL_2777478, EPI_ISL_2777479, EPI_ISL_2777481, EPI_ISL_2777482, EPI_ISL_2777483, EPI_ISL_2777484, EPI_ISL_2777485, EPI_ISL_2777995, EPI_ISL_2777996, EPI_ISL_2777997, EPI_ISL_2777998, EPI_ISL_2777999, EPI_ISL_2778000			
see above	Laboratorio de Ecologia de Doencas Transmissiveis na Amazonia, Instituto Leonidas e Maria Deane - Fiocruz Amazonia	Laboratorio de Ecologia de Doencas Transmissiveis na Amazonia, Instituto Leonidas e Maria Deane - Fiocruz Amazonia	André Corado; Debora Duarte; Felipe Naveca; Felipe Naveca on behalf of the Fiocruz COVID-19 Genomic Surveillance Network; Fernanda Nascimento; George Silva; Karina Pessoa; Luciana Gonçalves; Maria Júlia Brandão; Matilde Mejía; Michele Jesus; Valdinete Nascimento; Victor Souza; Ágatha Costa
EPI_ISL_2777236, EPI_ISL_2777238, EPI_ISL_2777248, EPI_ISL_2777249, EPI_ISL_2777250, EPI_ISL_2777251, EPI_ISL_2777320, EPI_ISL_2777495, EPI_ISL_2777498, EPI_ISL_2777499, EPI_ISL_2777503, EPI_ISL_2777507, EPI_ISL_2777508, EPI_ISL_2777509, EPI_ISL_2777516, EPI_ISL_2777539, EPI_ISL_2777599, EPI_ISL_2777698, EPI_ISL_2777700, EPI_ISL_2777701, EPI_ISL_2777740, EPI_ISL_2777986, EPI_ISL_2777987, EPI_ISL_2777993, EPI_ISL_2778002, EPI_ISL_2778004, EPI_ISL_2778005			
see above	Laboratório Central de Saúde Pública do Amazonas - LACEN-AM	Laboratorio de Ecologia de Doencas Transmissiveis na Amazonia, Instituto Leonidas e Maria Deane - Fiocruz Amazonia	André Corado; Debora Duarte; Felipe Naveca; Fernanda Nascimento; George Silva; Karina Pessoa; Luciana Gonçalves; Maria Júlia Brandão; Matilde Mejía; Michele Jesus; Valdinete Nascimento; Victor Souza; Ágatha Costa
EPI_ISL_434571	Microbiology, The University of Hong Kong	Microbiology, The University of Hong Kong	Chan; J.F.W. and Yuen; K.-Y.
EPI_ISL_539850, EPI_ISL_539851	Pok Oi Hospital	Hong Kong Department of Health	Alan K.L. Tsang; Dominic N.C. Tsang; Edman T.K. Lam; Peter C.W. Yip; Rickjason C.W. Chan
EPI_ISL_417064	Prince of Wales Hospital	Hong Kong Department of Health	Alan K.L. Tsang; Dominic N.C. Tsang; Edman T.K. Lam; Peter C.W. Yip; Rickjason C.W. Chan
EPI_ISL_539820	Queen Mary Hospital	Hong Kong Department of Health	Alan K.L. Tsang; Dominic N.C. Tsang; Edman T.K. Lam; Peter C.W. Yip; Rickjason C.W. Chan
EPI_ISL_417443	State Key Laboratory for Emerging Infectious Diseases Department of Microbiology Li Ka Shing Faculty of Medicine The University of Hong Kong	State Key Laboratory for Emerging Infectious Diseases Department of Microbiology Li Ka Shing Faculty of Medicine The University of Hong Kong	Bobo Wing-Yee Mok; Honglin Chen; Kwok-Yung Yuen; Pui Wang; Shaofeng Deng; Siu-Ying Lau; Wenjun Song
EPI_ISL_434570, EPI_ISL_450408, EPI_ISL_450409, EPI_ISL_450410, EPI_ISL_450411, EPI_ISL_450412	unknown	Microbiology	K.-Y.; K.K.W.; K.K.W. and Yuen; To; Yuen
EPI_ISL_434563, EPI_ISL_434564, EPI_ISL_434565, EPI_ISL_434566, EPI_ISL_434567, EPI_ISL_434568, EPI_ISL_434569			
see above	unknown	Microbiology, The University of Hong Kong	K.-Y.; K.K.W. and Yuen; To
EPI_ISL_450404, EPI_ISL_450405	unknown	School of Public Health, The University of Hong Kong	Brackman; C.J.; Chu; D.K.W.; D.N.C.; L.D.; L.L.M. and Peiris, M.; Perera; Poon; R.A.P.M.; Sims; Sit; T.H.S.; Tsang
